# The MEK inhibitor U0126 ameliorates diabetic cardiomyopathy by restricting XBP1's phosphorylation dependent SUMOylation

**DOI:** 10.7150/ijbs.60459

**Published:** 2021-07-13

**Authors:** Tao Wang, Jinhua Wu, Wei Dong, Mengwen Wang, Xiaodan Zhong, Wenjun Zhang, Lei Dai, Yang Xie, Yujian Liu, Xingwei He, Wanjun Liu, Thati Madhusudhan, Hesong Zeng, Hongjie Wang

**Affiliations:** 1 Division of Cardiology, Department of Internal Medicine, Tongji Hospital, Tongji Medical College, Huazhong University of Science and Technology, Wuhan, 430030, PR China; Hubei Key Laboratory of Genetics and Molecular Mechanisms of Cardiological Disorders, Wuhan, 430030, PR China; 2 Department of Cardiology, Affiliated Hospital of Weifang Medical University, Weifang, Shandong, 261000, PR China; 3 Departments of Respiratory and Critical Care Medicine, Guangdong Provincial People's Hospital, Guangzhou, 510000, PR China; 4 Hepatic Surgery Center, Tongji Hospital, Tongji Medical College, Huazhong University of Science and Technology, Wuhan, 430030, PR China; 5 Hubei Key Laboratory of Hepato-Pancreato-Biliary Diseases, Wuhan, Hubei, 430030, PR China; 6 Hubei Clinical Medicine Research Center of Hepatic Surgery, Wuhan, Hubei, 430030, PR China; 7 Key Laboratory of Organ Transplantation, Ministry of Education; NHC Key Laboratory of Organ Transplantation; Key Laboratory of Organ Transplantation, Chinese Academy of Medical Sciences, Wuhan, Hubei, 430030, PR China; 8 Center for Thrombosis and Hemostasis, University Medical Center Mainz, Langenbeckstr. 1, 55131 Mainz, Germany

**Keywords:** XBP1s, diabetic cardiomyopathy, endoplasmic reticulum stress, SUMOylation, U0126

## Abstract

**Background:** Chronic diabetes accelerates vascular dysfunction often resulting in cardiomyopathy but underlying mechanisms remain unclear. Recent studies have shown that the deregulated unfolded protein response (UPR) dependent on highly conserved IRE1α-spliced X-box- binding protein (XBP1s) and the resulting endoplasmic reticulum stress (ER-Stress) plays a crucial role in the occurrence and development of diabetic cardiomyopathy (DCM). In the present study, we determined whether targeting MAPK/ERK pathway using MEK inhibitor U0126 could ameliorate DCM by regulating IRE1α-XBP1s pathway.

**Method:** Three groups of 8-week-old C57/BL6J mice were studied: one group received saline injection as control (n=8) and two groups were made diabetic by streptozotocin (STZ) (n=10 each). 18 weeks after STZ injection and stable hyperglycemia, one group had saline treatment while the second group was treated with U0126 (1mg/kg/day), 8 weeks later, all groups were sacrificed. Cardiac function/histopathological changes were determined by echocardiogram examination, Millar catheter system, hematoxylin-eosin staining and western blot analysis. H9C2 cardiomyocytes were employed for *in vitro* studies.

**Results:** Echocardiographic, hemodynamic and histological data showed overt myocardial hypertrophy and worsened cardiac function in diabetic mice. Chronic diabetic milieu enhanced SUMOylation and impaired nuclear translocation of XBP1s. Intriguingly, U0126 treatment significantly ameliorated progression of DCM, and this protective effect was achieved through enriching XBP1s' nuclear accumulation. Mechanistically, U0126 inhibited XBP1s' phosphorylation on S348 and SUMOylation on K276 promoting XBP1s' nuclear translocation. Collectively, these results identify that MEK inhibition restores XBP1s-dependent UPR and protects against diabetes-induced cardiac remodeling.

**Conclusion:** The current study identifies previously unknown function of MEK/ERK pathway in regulation of ER-stress in DCM. U0126 could be a therapeutic target for the treatment of DCM.

## Background

Diabetes mellitus (DM) is one of the major chronic diseases endangering people's health worldwide. It has two major subtypes, type 1 (T1DM) and type 2 (T2DM). Cardiovascular disease (CVD) is a major cause of morbidity and mortality in diabetic patients. Patients with diabetes mellitus have a risk of cardiovascular events that is two to three times as high as the risk among those without diabetes^1^, ^2^. Interestingly, a subset of diabetic patients develops left ventricular dysfunction in the absence of coronary artery disease, hypertension or vascular disease. This observation, first made by Rubler *et al.* in 1972, is now known as diabetic cardiomyopathy (DCM)^3^. DCM is defined by the existence of abnormal myocardial structure and performance in the absence of other cardiac risk factors, such as coronary artery disease, hypertension, and significant valvular disease, in individuals with diabetes mellitus^4^. Their histopathological studies revealed diffuse fibrosis, myofibrillar hypertrophy, microvascular disease and deposition of acid mucopolysaccharide material^3^, ^4^.

With improvements in clinical treatments, the relative risk of death declined by 29% over a 10-year period, however, mortality in T1DM still increased 2- to 8- fold. Early onset T1DM is a strong risk factor associated with serious CVD, moreover, with a significant higher risk level in women, they also die around 18 years earlier than their diabetes free counterparts^5^-^8^. In compared with T2DM, several studies revealed that T1DM patients have an even higher incidence of heart failure (HF), when disease duration is >20 years, CVD becomes the primary cause of death^9^, ^10^. In addition, because of the incongruent clinical features the underlying mechanisms of CVD may be partially distinct in T1DM versus T2DM^11^. However, CVD in T2DM and T1DM is often considered the same disease, with the diagnosis and treatment of CVD in T1DM extrapolated from the experience with T2DM. Thus, it has become challenging to identify T1DM-specific mechanisms to develop T1DM-specific therapies for DCM^12^-^14^.

As reported in the literature, increases in oxidative stress, reduced nitric oxide bioavailability, elevations in advanced glycation end products, mitochondrial dysfunction and collagen-based cardiomyocyte and extracellular matrix stiffness, inflammation, renin-angiotensin-aldosterone system activation, cardiac autonomic neuropathy, endoplasmic reticulum (ER) stress, microvascular dysfunction and a myriad of cardiac metabolic abnormalities have all been implicated in the development and progression of DCM^4^, ^15^. Among these signaling pathways, ER stress pathway is continuously the studying focus and the three principle pathways of the unfolded protein response (UPR), including IRE1-XBP1, PERK-ATF4 and ATF6, are widely studied^16^, ^17^. XBP1, a key transcription factor of the UPR, is critical in maintaining ER homoeostasis and is activated by disturbances in endoplasmic reticulum (ER) protein-folding homeostasis. In response to upstream signals, XBP1 mRNA undergoes an unconventional splicing by the ER transmembrane endoribonuclease IRE1, which ultimately generates the potent transcriptional transactivator XBP1s. XBP1s promotes ER biogenesis and activates the expression of ER chaperone genes that are required for the folding and trafficking of secretory cargo proteins^18^. It plays a pivotal role in cardiac pathological regulation, such as cardiac ischemic reperfusion injury, transverse aortic constriction induced hypertrophy, *etc*. However, the XBP1s' role in DCM remains largely unknown^19^. Moreover, our previous study showed that the XBP1 signaling pathway is required for an adaptive ER-response in diabetic nephropathy (DN), while genetic disruption or functional inactivation of this pathway in diabetic mouse models promotes a maladaptive UPR. The latter is hallmarked by impaired spliced XBP1 (XBP1s) nuclear translocation, which provokes a maladaptive ER-response characterized by ATF6 and CHOP signaling in DN^20^. To this end, we proposed a vital role of XBP1s in cardiomyocytes in the context of maladaptive ER-response under DCM condition.

Furthermore, post-translational modification of XBP1, especially its SUMOylation has been shown to be involved in UPR regulation. Chen H. *et al.* reported that XBP1 can be SUMOylated by PIAS2 (protein inhibitor of activated STAT2) at two lysine residues located in the C-terminal transactivation domain. These SUMOylation events significantly decrease the transcriptional activity of XBP1 towards UPR target genes^21^. While others showed that Sentrin/SUMO-specific protease 1 (SENP1), a specific de-SUMOylation protease for XBP1 can increase the transcriptional activity of XBP1 and ER stress-induced apoptosis through accumulating SUMOylated XBP1^22^, ^23^. Thus, we speculated that SUMOylation of XBP1 may regulate UPR activation and ER homeostasis in DCM.

Within the current study, we confirmed a correlation of XBP1s' nuclear translocation in cardiomyocyte and DCM phenotype in a streptozotocin induced type 1 mouse DM model. Furthermore, we found that the impaired XBP1s' nuclear translocation is regulated by its SUMOylation in the diabetic hearts. Subsequently, the molecular mechanisms underlying the regulation of the XBP1s' SUMOylation in cardiomyocyte were systematically investigated, and we found that high glucose induced activation of MAP kinase ERK1/2 can increase XBP1s' phosphorylation on its serine residue S348, which in turn can promote its SUMOylation on lysine residue K276 and result in its impaired nuclear translocation. Finally, we tested whether the ERK1/2 inhibitor U0126 treatment can protect against DCM, as hypothesized U0126 treatment could be of therapeutic potential for DCM.

## Materials and methods

### Induction of diabetes using streptozotocin

Animal experiments were conducted following standards and procedures approved by the local Animal Care and Use Committee (Tongji Medical College Experimental Animal Center, Huazhong University of Science and Technology, China). Diabetes was induced by intraperitoneal administration of streptozotocin (STZ, Sigma-Aldrich, St. Louis, MO) at 60 mg /kg, freshly dissolved in 0.05 M sterile sodium citrate (pH 4.5) on five successive days in 8-week-old male C57BL/6J mice (HFK Bioscience, Beijing, China)^20^. Mice were considered diabetic if blood glucose levels determined from the tail vein using ONETOUCH glucose strips were above 300 mg/dl (16.7 mmol/L) after the last STZ injection. Mice displaying blood glucose levels above 500 mg/dl (27.7 mmol/L) received insulin Lantus (1-2U, Sanofi, Beijing, China) to avoid excessive and potentially lethal hyperglycemia. We injected a subset of diabetic mice intraperitoneally with either U0126 (LC Laboratories, Woburn, MA) (1mg/kg, dissolved in 6% DMSO-PBS) or 6% DMSO-PBS once daily starting 18 weeks after the last STZ injection until 1 day before analysis 20. Blood and tissue samples were obtained at 26 weeks after injection of STZ.

### Echocardiographic analysis

Mice were anesthetized using light isoflurane (1-2%). Echocardiography was performed as described previously using a high-resolution imaging system with a 13-MHz linear ultrasound transducer (VisualSonics Vevo770, VisualSonics Inc., Toronto, Canada).

### Hemodynamic measurements of left ventricular (LV) function

Measurements of LV function were performed using Millar catheter system as described before. In brief, mice were anaesthetized, and a pressure-volume catheter (Millar 1.4F, SPR 835, Millar Instruments, Inc. Houston, TX, USA) was inserted into the right carotid artery and advanced into the left ventricle under pressure control to measure instantaneous intraventricular pressure and volume.

### Histology and immunohistochemistry

Histology analysis was performed as previously described^20^, ^24^. We first perfused freshly sacrificed mice with ice-cold PBS and then with 4% paraformaldehyde. Tissues were further fixed in 4% paraformaldehyde for 48 hours, embedded in paraffin and processed for sectioning. Cardiomyocyte area was quantified in sections stained with H&E (JianCheng, Nanjing, China) and wheat germ agglutinin (WGA) (Vector Laboratories, Burlingame, CA). Images were also captured with a Nikon-Microscope (NIKON, Tokyo, Japan). The outline of myocytes was traced using Image J software to determine myocyte cross-sectional area in the LV of each animal. A value from each heart was calculated by measuring about 400-600 cells in a remote area from 5 randomly selected image areas in an individual heart.

### Cell culture and transfection

H9C2 cells (ATCC) were cultured in RPMI 1640 medium (Sigma-Aldrich, St. Louis, MO) supplemented with 10% Fetal bovine serum (FBS, Life Technologies, UK) and Penicillin/Streptomycin (P/S) (100 IU/mL) in a humidified atmosphere of 95% air and 5% CO2 at 37°C. HEK293T cells (ATCC) were maintained in DMEM medium supplemented with 10% FBS. Cells were starved overnight before treatment with high concentrations of glucose (HG, 25 mM) or mannitol (25 mM). Transfection was performed using transfection reagent FuGENE (Promega, Germany) according to the manufacturer's protocol. At desired time points post glucose and mannitol treatment, total lysates or cytosolic and nuclear fractions of cells were prepared for immunoblotting analysis.

### Cell fractionation

Isolation of cytosolic and nuclear fractions was performed as previously described^20^. Heart samples were lysed using tissue homogenizer in buffer-A containing 10 mM HEPES-KOH (pH 7.9), 10 mM KCl, 1.5 mM MgCl_2_, 1 mM EDTA, 0.6% NP-40, 0.5 mM DTT, protease inhibitor cocktail (Roche diagnostics GmbH, Mannheim, Germany) and lysates were incubated for 10 min on ice. After brief vortexing the lysates were centrifuged for 10 min at 5,000 rpm at 4°C. Supernatants were collected as cytosolic fractions and the pellets were resuspended in 100 ml of buffer-B containing 10 mM HEPES-KOH (pH 7.9), 25% glycerol, 420 mM NaCl, 1.5 mM MgCl_2_, 0.2 mM EDTA, 0.5 mM DTT and protease inhibitors. Lysates were incubated for 20 min on ice followed by centrifugation at 12,000 g at 4°C for 10 min. Supernatants containing the nuclear extracts were collected and stored at - 80°C.

A similar procedure was used for isolation of cytosolic and nuclear fractions of cells where buffer-A contains 10 mM HEPES-KOH (pH 7.9), 10 mM KCl, 1.5 mM MgCl_2_, 0.5 mM DTT and protease inhibitors. Protein concentration was measured using Bradford reagent or BCA reagent (Boster, Wuhan, China), and purity of nuclear and cytoplasmic fractions was determined by Lamin A/C (Santa Cruz Biotechnology, Inc) and GAPDH (Cell Signaling Technology, Danvers, MA) western blots, respectively.

### Immunoblotting and Immunoprecipitation

Immunoblotting and Immunoprecipitation were performed as previously described^20^, ^25^. In brief, for immunoblotting total lysates were prepared in RIPA buffer (50 mM Tris at pH 7.4, 1% NP-40, 0.25% sodium deoxycholate, 150 mM NaCl, 1 mM EDTA, and 1 mM Na_3_VO_4_, supplemented with protease inhibitor cocktail). Lysates were centrifuged (12,000 × g for 20 min at 4 °C) and supernatant was kept, while the pellet containing debris was discarded. The protein concentration in supernatants was quantified using BCA reagent. Equal amounts of protein were electrophoretically separated on 10% or 12.5% SDS polyacrylamide gels, transferred to PVDF membranes, and probed with the desired primary antibodies overnight at 4 °C. Membranes were then washed with TBST and incubated with anti-mouse or anti-rabbit IgG (1:2000, Abcam, Cambridge, MA) horseradish peroxidase-conjugated antibodies as indicated. Blots were developed with the immobilon western chemiluminiscent HRP substrate. The density of each band was measured by using ImageJ software.

For Immunoprecipitation of total cellular and heart tissue proteins, proteins were extracted with RIPA containing complete protease inhibitor cocktail. Lysates were combined with 1 μg of specific antibody and incubated overnight at 4 °C on rotating shaker. Immunoprecipitates were purified with protein A/G agarose beads (Santa Cruz, CA) and washed with PBS containing protease inhibitor cocktail. Immunoprecipitates were fractionated by SDS-PAGE (10%), transferred to PVDF membranes, and subjected to immunoblotting with appropriate primary and secondary antibodies as described above. The following antibodies were used in the current study: ANP (Thermo Fisher Scientific, Rockford, USA), Flag, XBP-1, BNP (Santa Cruz Biotechnology, Inc), SUMO-1, SUMO2/3, ERK1/2, P-ERK1/2, P38, p-P38, JNK, p-JNK (Cell Signaling Technology, Danvers, MA), β-MHC (Abcam, Cambridge, MA).

### Plasmid DNA, Adenovirus and Quick-change mutagenesis

Plasmids encoding wildtype or lysine mutant XBP1s-GFP fusion (K276R, K297R and K298R XBP1s) proteins were gifts from Dr. Ling Qi (Cornell University)^21^. Mouse XBP1s mutations (Ser61Ala, Ser169Ala, and Ser348Ala) were achieved using the Quick Change Site-Directed Mutagenesis Kit (Stratagene, La Jolla, CA) with the following primers respectively: Ser61Ala: 3'-GCTCACGCACCTGGCCCCGGAGGAGAAAG-5' (F), 5'-CTTTCTCCTCCGGGGCCAGGTGCGTGAGC'-3(R); Ser169Ala: 3'-GCCCAGTTGTCACCGCCCCAGAACATCTTC-5' (F), 5'-GAAGATGTTCTGGGGCGGTGACAACTGGGC-3'(R); Ser348Ala: 3'-CTTCAGTGACATGTCTGCTCCACTTGGTACAG-5' (F), 5'-CTGTACCAAGTGGAGCAGACATGTCACTGAAG-3' (R); All mutants were confirmed by sequencing. Ad-EGFP, Ad-XBP1s, Ad-K276R XBP1s, Ad-K297R XBP1s, Ad-K298R XBP1s and Ad-S348A XBP1s (Shanghai DesignGene Biotechnology Co., Ltd) were carried out in H9C2 cells for 24 hours. Adenoviruses were transduced at 25 multiplicity of infection (MOI).

### Real Time PCR

To examine the downstream UPR genes in wild type and K276R mutant transfected H9C2 cells, we performed UPR pathway-specific expression analyses. Quantitative RT-PCR were conducted essentially as previously described^26^. Transfected H9C2 cells were thawed on ice and transferred into TRIZOL (Life Technologies, Shanghai, China) for isolation of total RNA following the manufacturer's protocol. Quality of total RNA was ensured on an agarose gel and by analyses of the A260/280 ratio. The reverse transcription reaction was conducted with 1 μg of total RNA using the Super Script reagents and oligo (dT) primers (Sangon Biotech, Shanghai, China). cDNA was amplified using the primers listed in the Supplementary Table S1.

### Immunofluorescence

Fluorescent microscopy was performed as previously described^21^. H9C2 cells split onto sterile cover glasses coated with poly-L-lysine (Sigma) were transfected with adenoviruses encoding wildtype or lysine mutant XBP1s-GFP fusion proteins and then incubated with HG for 6 hours. Cells cover glasses were removed from dishes and then counterstained with DAPI. The fluorescent images were taken with a Nikon Eclipse E600 microscope or a Zeiss Axiovert 135.

Immunofluorescence microscopy was performed as previously described^27^. Briefly, H9C2 cells on coverslips were fixed with 4% paraformaldehyde for 15 min, then washed with PBS three times. The cells were blocked with normal goat serum and incubated with anti-XBP1s antibody overnight. Cells were washed with PBS three times and then incubated with secondary antibodies conjugated with Alexa Fluor 488 goat anti-mouse IgG antibody (Invitrogen) for 1 hour. Cells were washed with PBS three times and then counterstained with DAPI. Immunofluorescence images were taken with a Nikon Eclipse E600 microscope or a Zeiss Axiovert 135.

### Statistical analysis

The data are summarized as the means ± SEM (standard error of the mean). Statistical analyses were performed with Student's *t*-test or ANOVA as appropriate and post-hoc comparisons of ANOVA were corrected with the method of Tukey. Prism 5 (www.graphpad.com) software was used for statistical analyses. All data presented involving cell culture is representative of at least three independent repeat experiments. Statistical significance was accepted at values of *p* < 0.05.

## Results

### Diabetic mice displayed cardiac function impairment and left ventricular hypertrophy

**Figure 1:**
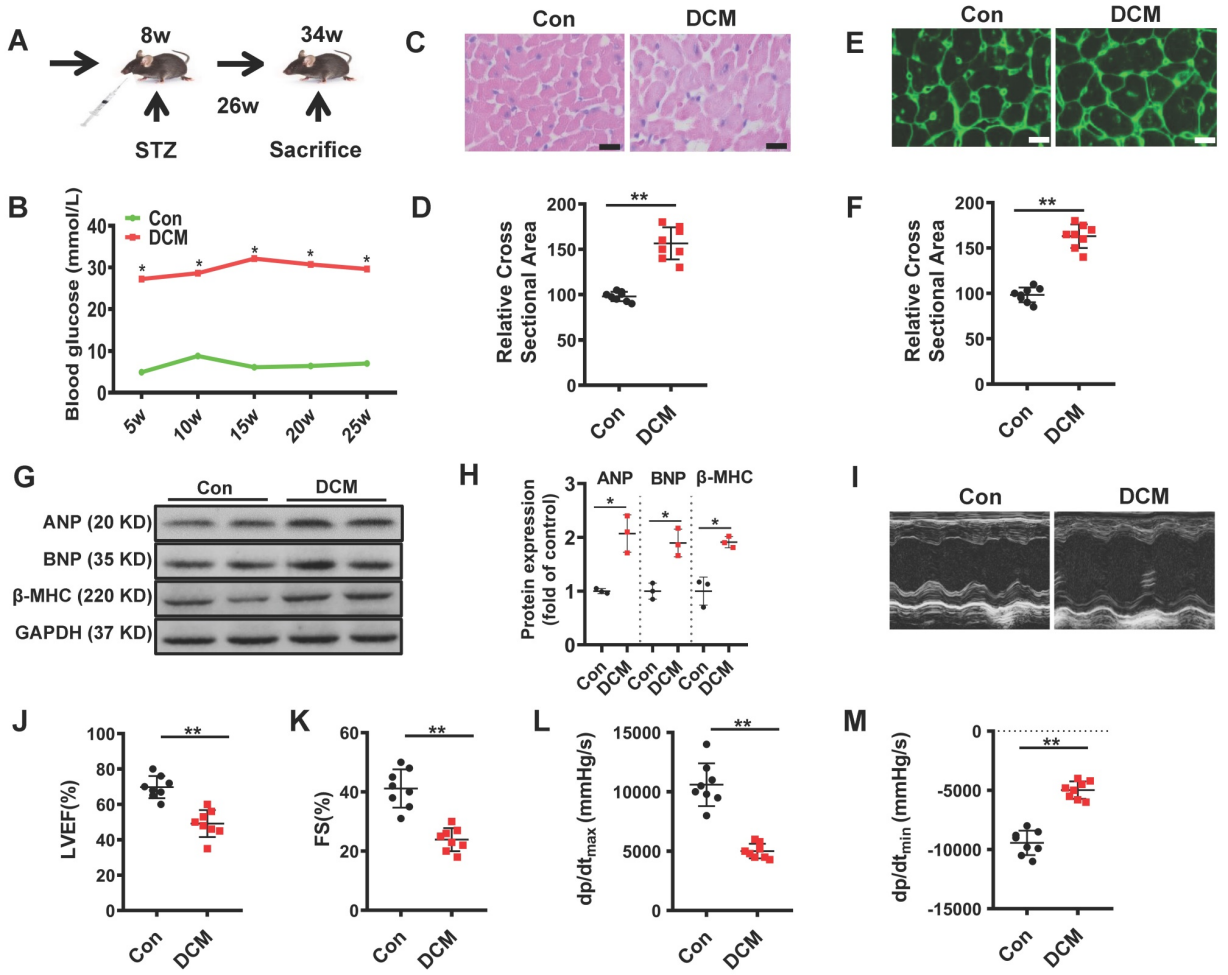
** Establishment of diabetic cardiomyopathy mouse model. (A)** schematic illustration of interventional studies in mice with STZ-induced hyperglycemia. **(B)** Line graphs reflecting blood glucose levels in mice with STZ-induced hyperglycemia. Blood glucose was measured at indicated time points. Histological analyses of the H&E staining and WGA staining of Con and DCM mice 26 weeks after STZ injection. **(C)** Representative images of H&E staining in the hearts of Con and DCM mice (n ≥ 8; scale bar, 100 μm). **(D)** Statistical results of the myocyte cross-sectional areas (n ≥ 6, at least 100 cells per mouse were analyzed). **(E)** Representative images of WGA staining in the hearts of Con and DCM mice (n ≥ 8; scale bar, 100 μm). **(F)** Quantitative analysis of myocyte cross-sectional areas (n ≥ 6, at least 100 cells per mouse were analyzed). **(G-H)** Representative immunoblots of Atrial natriuretic peptide (ANP), B type natriuretic peptide (BNP) and β-myosin heavy chain (MHC) expression and quantitative analysis are shown. **(I-K)** Echocardiographic measurements of ventricular functional parameters includes: Ejection fraction **(J)** and fractional shortening** (K)**. Representative echocardiographic M-mode images** (I)**. **(L-M)** Cardiac haemodynamic measurements including +dP/dt_max_ and -dP/dt_min_ were also conducted. Con, control mice without diabetes, black dots; DCM, diabetic cardiomyopathy, red squares. Data are shown as mean ± SEM (n ≥ 6 per group); *p < 0.05, **p < 0.01 *vs* Con group. H&E, hematoxylin eosin; WGA, wheat germ agglutinin. LVEF, left ventricle ejection fraction; FS, fractional shortening; +dp/dt_max_, the maximal rate of pressure development; -dp/dt_min_, the minimal rate of pressure decay.

As described in materials and methods, we used a mouse model of persistent hyperglycemia induced by streptozotocin (STZ model), reflecting insulinopenic type 1 DM **(Figure 1A)**^28^. After established diabetes, we detected random blood glucose in the indicated time points. Random blood glucose levels were significantly increased in STZ-induced mice compared with the control group **(Figure 1B)**. As shown in **Figure 1C, E**, the myocardial structure was examined by Hematoxylin and eosin (H&E) and WGA staining, diabetic hearts displayed structural abnormalities, including disrupted cardiac fibers, deranged cellular structures, obscured intercellular border and increased cardiomyocyte transverse cross-sectional areas (**Figure 1D, F**). Immunoblots showed an increase in the expression of atrial natriuretic peptide (ANP), B type natriuretic peptide (BNP) and β-myosin heavy chain (β-MHC) in the heart tissues of diabetic cardiomyopathy mice (**Figure 1G-H**). To evaluate the changes of left ventricular performance in diabetic mice, M-mode echocardiography was performed at 26 weeks after STZ injection **(Figure 1I)**. Compared to control, diabetic cardiomyopathy mice showed a significant reduction of ejection fraction and fraction shorting** (Figure 1J-K)**. We also detected the left ventricular hemodynamic alternation simultaneously. Diabetic mice showed aggravated cardiac dysfunction, which was mainly manifested in the decline in + dP/dt _max_ and - dP/dt _min_ **(Figure 1L-M)**. These data indicated that the type 1 DCM was successfully established, characterized by diastolic and systolic dysfunction and myocardial hypertrophy.

### XBP1s' nuclear translocation was significantly reduced and negatively correlated with the level of its SUMOylation in diabetic mouse heart

**Figure 2:**
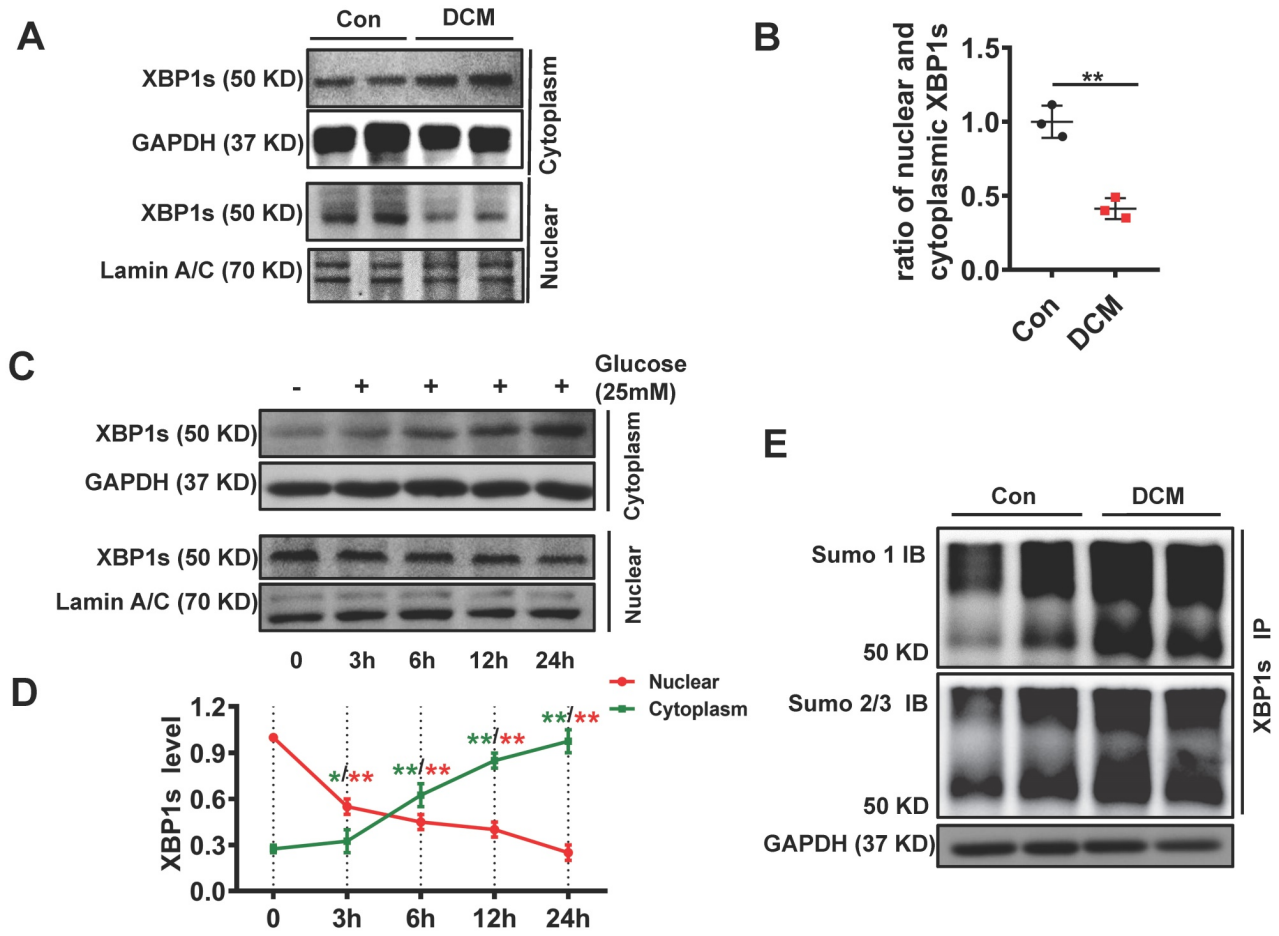
** XBP1s' nuclear translocation was significantly reduced and negatively correlated with the level of its SUMOylation in STZ-induced diabetic mouse heart.** XBP1s nuclear translocation is impaired in DCM mice and in H9C2 cardiomyocytes treated with HG (25mM). **(A-D)** Representative immunoblots **(A)** and bar graph **(B)** showing nuclear and cytoplasm levels of XBP1s in heart samples of wild-type control (Con) and STZ-induced diabetic cardiomyopathy mice (DCM). As loading controls, Lamin A/C was used for nuclear extracts and GAPDH for cytoplasm extracts (n=6 mice per group). **(C-D)** Representative immunoblots showing cytoplasmic (C, top panel) and nuclear levels (C, lower panel) of XBP1s in H9C2 cardiomyocytes at indicated time points after treatment with HG (25mM). Line graph **(D)** reflecting the Mean ± SEM of three independent experiments. **(E)** XBP1s' SUMOylation was increased in DCM group. Heart samples from Con and DCM mice were immunoprecipitated with anti-XBP1s antibody, and Western blot analysis was performed with anti-SUMO1 (top panel) and anti-SUMO2/3 (lower panel) antibodies. A significant increase in XBP1s' SUMOylation was observed in DCM group. Con, control mice without diabetes, black dots; DCM, diabetic cardiomyopathy, red squares; IB, immunoblot; IP, immunoprecipitation. Mean ± SEM, (B, D); ** p < 0.01 *vs* Con group. * p < 0.05 *vs* 0 h; ** p < 0.01 *vs* 0 h (B: *t*-test; D: ANOVA); Representative immunoblots of at least three independent experiments (A, C and E).

Chronic hyperglycemia in diabetes impairs nuclear translocation of XBP1s compromising UPR resulting in maladaptive ER-stress in diabetic kidney^20^, ^29^. In agreement with these observations, chronic diabetes induced impaired nuclear translocation of XBP1s in the diabetic heart. **(Figure 2A, B)**. Besides, other brunches of the UPR were subsequently deregulated, such as ATF6 and CHOP (**Supplementary Figure 1**). Furthermore, *in vitro* studies with neonatal cardiomyocytes H9C2 cells showed that high concentration glucose (HG, 25mM) but not mannitol impaired nuclear translocation of XBP1s **(Figure 2C-D, Supplementary Figure 2)**. These data suggest that akin to diabetic kidney, nuclear translocation of XBP1s is impaired in diabetic heart promoting maladaptive ER-stress and cardiomyopathy.

SUMOylation of proteins, post-translational modification by the small ubiquitin-like modifier (SUMO), is an important transient regulatory mechanism in many cellular processes, most notably transcriptional regulation, DNA damage, and signal transduction^30^, ^31^. To identify whether SUMOylation of XBP1s modulates its protective functions in DCM, we conducted immunoprecipitation for total proteins extracted from cardiac tissue of control and DCM mice. We found that XBP1s protein was SUMOylated by SUMO1 and SUMO2/3 in DCM mice **(Figure 2E, Supplementary Figure 3)**. Taken together, these data suggest that its SUMOylation could regulate the impaired XBP1s nuclear translocation observed in STZ-induced diabetic cardiomyopathy mice.

### HG inhibits XBP1s' nuclear translocation through SUMOylation of XBP1s at the lysine residue 276

**Figure 3:**
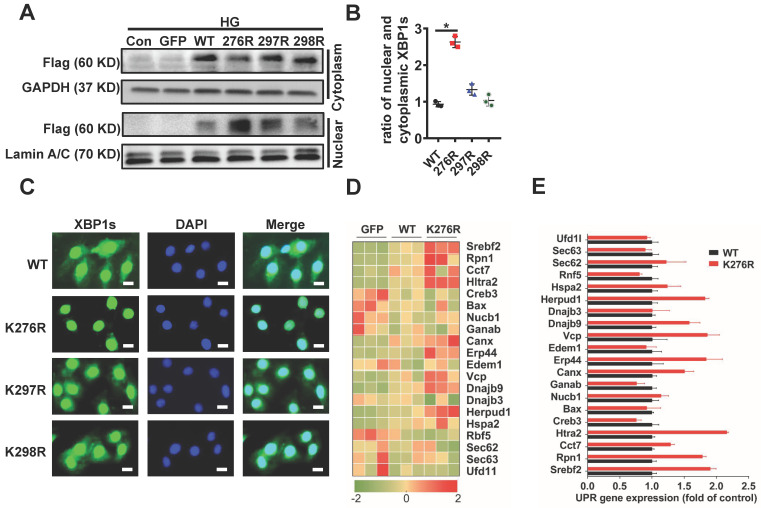
** SUMOylation of XBP1s at K276 residue at the C-terminal transactivation domain reduced its nuclear localization under HG condition. (A-B)** Immunoblots and bar graph of Flag-XBP1s from Nuclear and cytoplasmic protein extracts in H9C2 cells upon transfection with different Flag-XBP1s constructs. As loading controls, Lamin A/C was used for nuclear extracts and GAPDH for cytoplasm extracts. **(C)** Fluorescent microscopic images of XBP1s-GFP fusion constructs upon 24 h transfection into H9C2 cells following HG intervention for 6 h. Nuclei were counter-stained with DAPI. Scar bar: 25μm. **(D)** Heat map summarizes enrichment of condition-specific concordant UPR genes. **(E)** And their impact on regulation of UPR-gene expression (E; n=3; Mean ± SEM, wild type *vs* K276R mutant).

SUMOylation of transcription factors often leads to alterations in the intracellular localization of target proteins^30^. Therefore, we investigated the physiological significance of SUMOylation on XBP1s' nuclear translocation. Lysine residues K276 and K297 were identified as two highly-conserved SUMOylation motifs and located within the transactivation domain of the XBP1s protein, a region that is absent in the XBP1u (unspliced XBP1) protein^21^. This sequence is highly conserved across the different paralogous homologs in mouse, human, rat and other species** (Supplementary Figure 4A)**. In order to exclude the possibility of non-specific modification events, mutation of K298 to R (K298R) which had no effect on SUMOylation of XBP1s protein that carried a negative charge. The scheme shown in **Supplementary Figure 4A** summarizes mutational loci of K276R, K297R and K298R. Next, we expressed XBP1s-GFP fusion proteins for wild type and lysine mutant XBP1s-GFP fusion (K276R, K297R and K298R) proteins in H9C2 cells **(Supplementary Figure 4B-C)**. Interestingly, mutation of K276 to R (K276R) obviously increased XBP1s' nuclear translocation, while other mutations had no significant effect **(Figure 3A, B)**. Consistent with this result, fluorescent microscopic images of XBP1s-GFP fusion adenovirus constructs upon 24 hours transfection into H9C2 cells following HG intervention for 6 hours showed that mutant K276R predominantly localized to the nucleus **(Figure 3C)**. To further prove its functional relevance in ER-stress, we examined the downstream UPR genes in wild type and K276R mutant transfected H9C2 cells simultaneously. In congruent with increased nuclear translocation of XBP1s, when compared to wild type, K276R mutant enhanced the expression of genes that modulate UPR **(Figure 3D-E)**. Thus, our data suggest that HG inhibited XBP1s' nuclear translocation through enhancing its SUMOylation at lysine residue 276.

### MAPKs are differentially regulated in diabetic mouse heart

**Figure 4:**
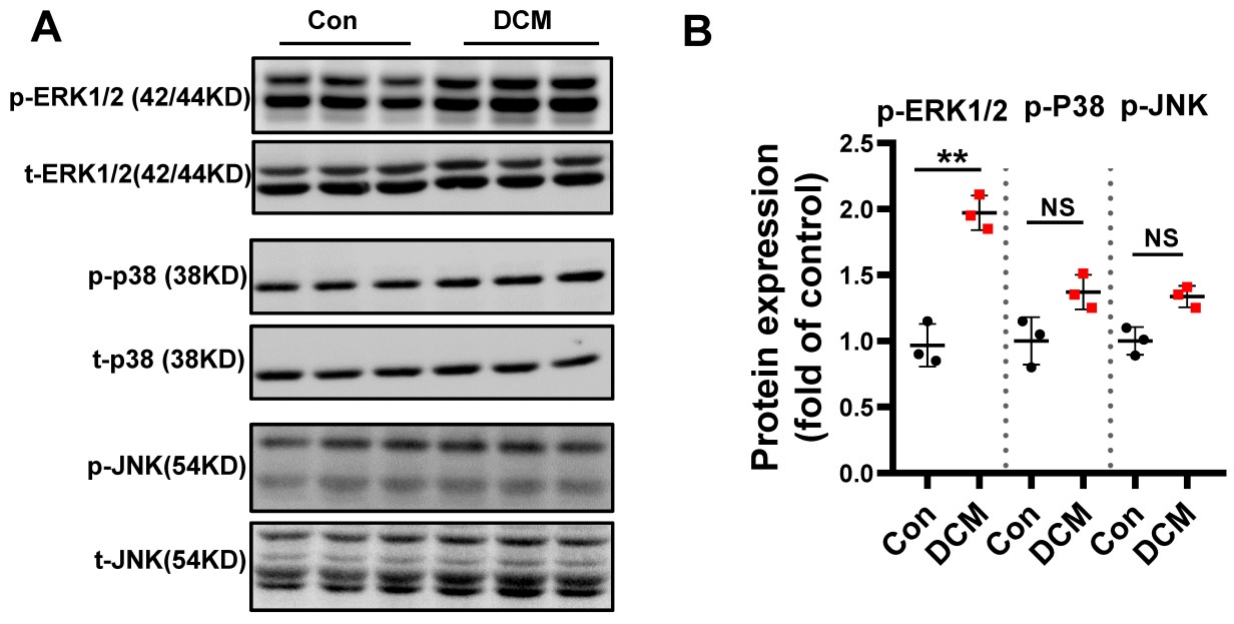
** Differential regulation of MAPK kinase in STZ-induced diabetic mouse heart. (A)** Western blot analysis of protein extracts from hearts in different mice as indicated using specific antibodies against the phosphorylated and the non-phosphorylated form of the MAPKs ERK1/2, p38, JNK and **(B)** scatter blot summarized the results. We could show an increase in the phosphorylated form of the MAPKs ERK1/2 but not in p38 and JNK. Con, control mice without diabetes, black dots; DCM, diabetic cardiomyopathy, red squares; Data represent ratios of densitometric measurements of either p-JNK or JNK, p-ERK and ERK1/2 or p-p38 and p38 and are presented as Mean ± SEM. **P < 0.01 *vs* Con group; NS, non-significant (*t*-test). Representative immunoblots of at least three independent experiments (A).

Mitogen-activated protein kinases (MAPKs) consist of three subfamilies, including extracellular signal-regulated kinase 1/2 (ERK1/2), p38, and c-Jun N-terminal kinase (JNK)^32^. A large number of *in vivo* and *in vitro* studies have demonstrated that chronic hyperglycemia can activate MAPKs, which play a pivotal role in the development of DCM^33^. To assess MAPK signaling activity, levels of total and phosphorylated ERK1/2, JNK, and p38 in diabetic hearts were determined. We found that the expression of phosphorylated ERK1/2 was significantly increased in diabetic mouse heart when compared to non-diabetic control heart samples. However, the levels of phosphorylated p-JNK and p-p38 were not significantly induced (**Figure 4A-B**). Therefore, these data indicated ERK1/2 could be one of the important effectors in response to consistent hyperglycemia.

### Phosphorylation dependent SUMOylation of XBP1s is triggered by HG-induced ERK1/2 activation

**Figure 5:**
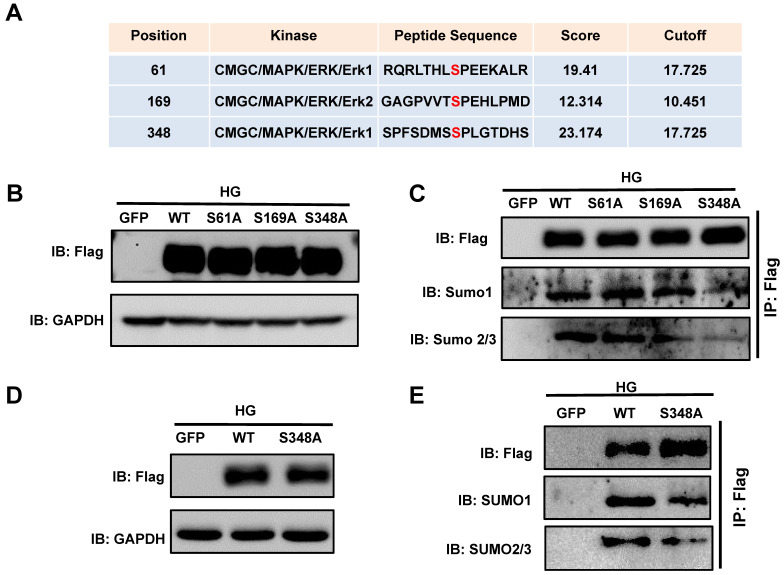
** Predicting and validating ERK1/2 Serine phosphorylation sites on XBP1s. (A)** Potential phosphorylated Serine sites on XBP1s by ERK1/2 were predicted by the phosphorylation prediction software GPS 3.0. **(B)** Plasmid wild type XBP1s (WT) and serine-to-alanine phospho-mutants of XBP1s (S61A, S169A and S348A) were expressed in HEK293T cells and lysates were immunoblotted using the indicated antibodies. **(C)**Additionally, lysates were immunoprecipitated (IP) using Flag-agarose and immunoblotted using the indicated antibodies. **(D)** H9C2 cells were infected with wild type and XBP1s S348A mutant carrying adenoviruses, lysates were immunoblotted using the indicated antibodies. **(E)** Subsequently, lysates were immunoprecipitated (IP) using Flag-agarose and immunoblotted using the indicated antibodies. Results are representative of three independent experiments (B-E).

Given the specific increase in p-ERK1/2 in the diabetic heart, we next determined whether the phosphorylation of ERK1/2 modulates XBP1s function by modulating its phosphorylation. For this we investigated the ERK1/2 phosphorylation sites on XBP1s. By using the phosphorylation prediction software GPS 3.0 (http://gps.biocuckoo.org/userguide.php), we identified a series of potential ERK1/2 phosphorylation sites on XBP1s and the three highest-ranking sites (Ser61, Ser169, Ser348; **Figure 5A**) were selected. We performed site-directed mutagenesis with Alanine (Ala) to analyze the functional relevance of these phosphorylation sites.

Phosphorylation is an important protein posttranslational modification^34^ that modulates additional post-translational modifications, including ubiquitination, SUMOylation, and acetylation, and in turn nuclear localization of proteins^35^. To determine whether XBP1s phosphorylation regulated its SUMOylation, we transiently transfected HEK293T cells with the Flag-XBP1s-WT and phospho-mutant XBP1s plasmids (S61A, S169A, S348A). The cells were subsequently exposed to HG for 6 hours. Immunoblot results showed that all mutants were functional and equally expressed (**Figure 5B**). Immunoprecipitation was performed using a Flag antibody. As shown in **Figure 5C**, the SUMO1 and SUMO2/3 levels were significantly decreased, indicating that mutation of the ERK consensus site (Ser 348) in XBP1s has a substantial effect on XBP1s' phosphorylation and SUMOylation. However, The S69A and S161A mutants showed no decreases in XBP1s SUMOylation **(Figure 5C)**, indicating that the Ser348 site has a crucial role in XBP1s SUMOylation.

To ascertain whether the phosphorylation of XBP1s at the Ser348 site by ERK1/2 regulate its SUMOylation in H9C2 cells, we constructed adenoviruses carrying the XBP1s wild type and S348A mutant and expressed in H9C2 cells. As shown in **Figure 5D-E**, consistent with the foregoing results, the SUMO1 and SUMO2/3 levels were significantly reduced in S348A mutant in compared with WT construct. These data indicated that ERK1/2 pathway regulates HG-induced XBP1s SUMOylation by phosphorylating Ser348.

### ERK1/2 inhibition increases XBP1s' nuclear translocation by inhibiting XBP1s' SUMOylation in H9C2 cells *in vitro*

ERK1/2 signaling pathway could promote cardiac differentiation and plays a role in myocardial protection in myocardial ischemia reperfusion^36^, ^37^. Additionally, MEK1/2 inhibitor U0126 attenuates ischemia/reperfusion-induced apoptosis and autophagy in myocardium^38^ and attenuates cisplatin-induced renal injury by decreasing inflammation and apoptosis by inhibition of ERK1/2 phosphorylation^39^. In agreement with these data, ERK1/2 phosphorylation was increased in diabetic mouse heart (**Figure 4A-B**) and ERK1/2 pathway regulated HG-induced XBP1s phosphorylation and SUMOylation (**Figure 5A-E**). So, we used U0126, an inhibitor of MEK1/2, for subsequent experimental studies.

**Figure 6:**
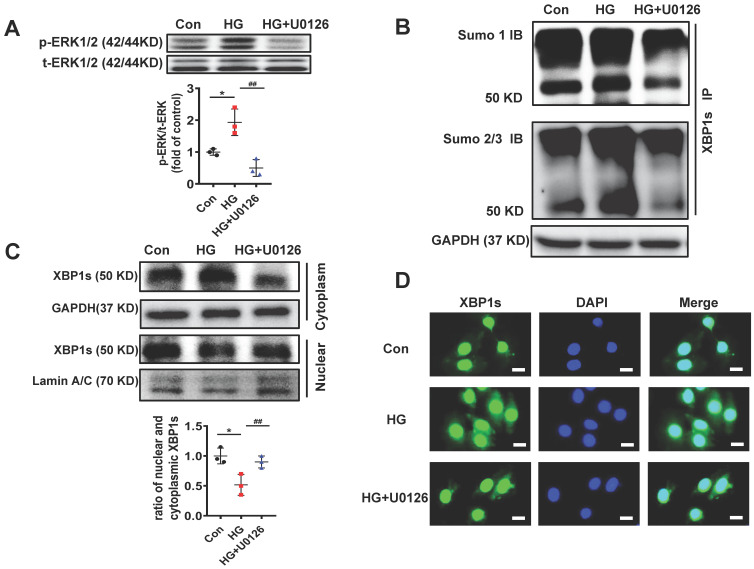
** U0126 increased XBP1s' nuclear translocation through inhibiting its SUMOylation in HG-treated H9C2 cells *in vitro*. (A)** Expression of total-ERK1/2 (t-ERK1/2) and phospho-ERK1/2 (p-ERK1/2) was detected by Western blot after H9C2 cells pretreated with U0126 (10 μM) for 1 h and HG (25mM) for additional 6 h. Representative immunoblots (top panel) and scatter blot (lower panel) summarizing results. **(B)** Extracted total proteins were immunoprecipitated with anti-XBP1s antibody, and Western blot analysis was performed with anti-SUMO1 (top panel) and anti-SUMO2/3 (lower panel) antibodies. A significant increase in XBP1s-SUMOylation was observed in HG group.** (C)** Representative immunoblots (top panel) and scatter blot (lower panel) showing cytoplasm and nuclear levels of XBP1s in the extracted total proteins of Con, HG and HG + U0126 group. As loading controls, Lamin A/C was used for nuclear extracts and GAPDH for cytoplasm extracts. **(D)** In the same experimental setting immunofluorescence staining was conducted, XBP1s was stained with green color, while nuclei were counter stained with DAPI (blue color). Scar bar: 25μm.Con, control without HG, black dots; HG: high glucose, squares; U0126, blue triangles; IB, immunoblot; IP, immunoprecipitation. Mean ± SEM (A, B); *P < 0.05 *vs* Con group, ^##^*P* < 0.01 *vs* HG group (A, B: ANOVA); Representative IBs of at least three independent experiments (A, B and C).

We initially determined the efficacy of U0126 on the phosphorylation of ERK1/2 (p-ERK1/2). H9C2 cells pretreated with 10μM U0126 for 1 hour were incubated with HG (25mM) for additional 6 hours. As shown in **Figure 6A**, U0126 markedly reduced p-ERK1/2 level induced by HG.

To determine if the reduction of p-ERK1/2 by U0126 can regulate XBP1s' SUMOylation, we initially determined XBP1s SUMOylation in H9C2 cells in response to U0126 treatment. The results demonstrate that U0126 significantly inhibit XBP1 SUMOylation induced by HG in H9C2 cells **(Figure 6B)**. These data suggest that XBP1s SUMOylation is dependent on ERK phosphorylation *in vitro*.

Similar to increasing XBP1s' SUMOylation, U0126 promotes XBP1s' nuclear translocation, which is inhibited by HG in H9C2 cells **(Figure 6C-D)**. Taken together, ERK1/2 inhibition with U0126 can increase XBP1s' nuclear translocation by inhibiting XBP1s' SUMOylation in HG-induced H9C2 cells *in vitro*.

### ERK1/2 inhibition protects against DCM in mouse model of type 1 diabetes

**Figure 7:**
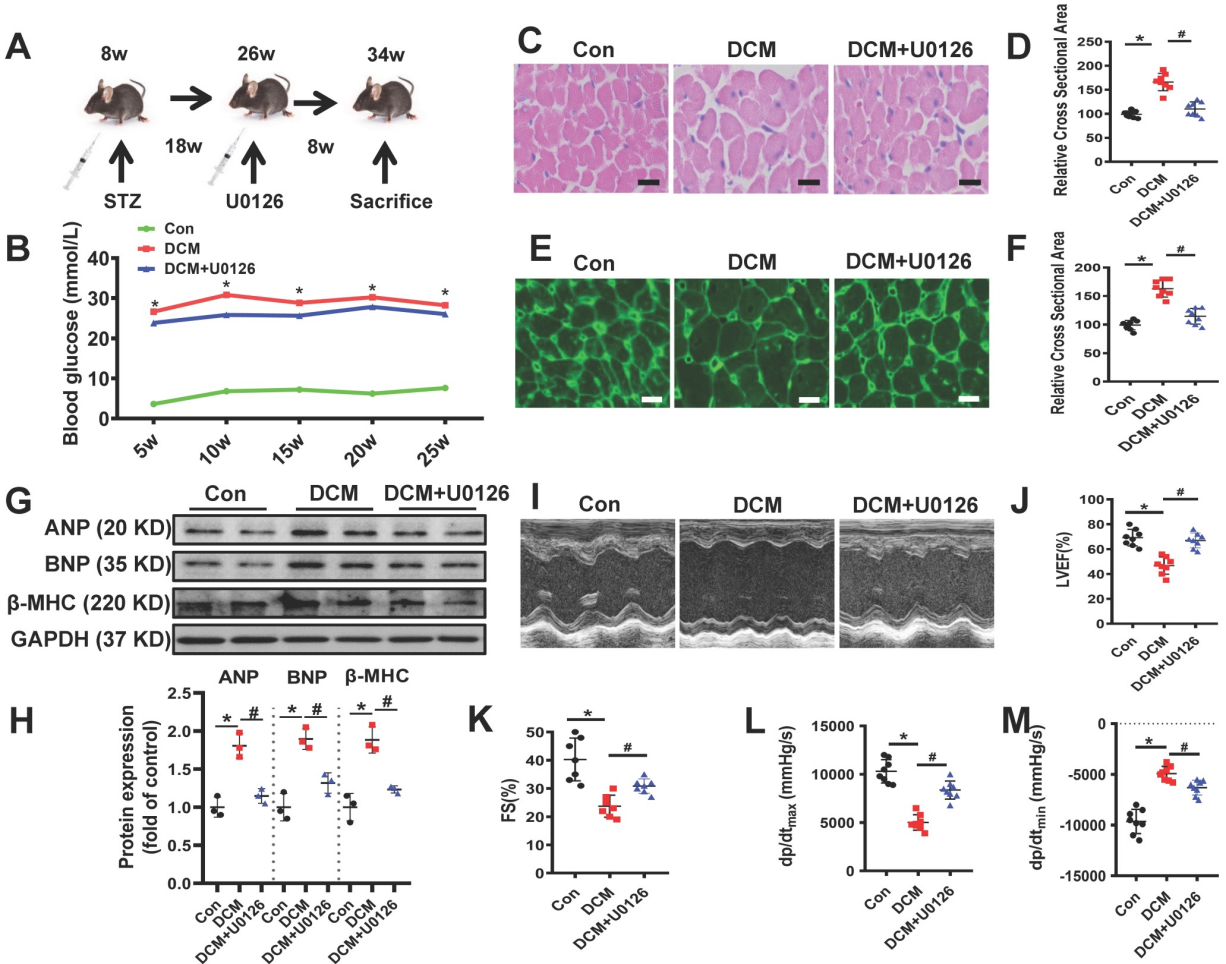
** U0126 improved cardiac function and ameliorated cardiac hypertrophy in STZ-induced diabetic mice. (A)** Schematic illustration of interventional studies in mice with STZ-induced hyperglycemia. Treatment with U0126 for 8 weeks was initiated after manifestation of abnormal cardiac function at week 18. **(B)** Line graphs reflecting blood glucose levels in mice with STZ-induced hyperglycemia. Blood glucose was measured at indicated time points. Histological analyses of the H&E staining and WGA staining of hearts from different mice as indicated 26 weeks after STZ injection. **(C)** Representative images of H&E staining in the hearts of each group mice (n ≥ 8; scale bar, 100 μm).** (D)** Statistical results of the myocyte cross-sectional areas (n ≥ 6, at least 100 cells per mouse were analyzed). **(E)** Representative images of WGA staining in the hearts of each group mice (n ≥ 8; scale bar, 100 μm). **(F)** Quantitative analysis of myocyte cross-sectional areas (n ≥ 6, at least 100 cells per mouse were analyzed). Representative immunoblots **(G)** and scatter blots summarizing results **(H)** for cardiac hypertrophy-related proteins expression in hearts from different mice as indicated. **(I)** Representative echocardiographic images of the left ventricle in different mice as indicated; **(J-K)** Echocardiographic assessment of LVEF and FS in different mice as indicated. **(L-M)** Cardiac hemodynamic measurements including +dP/dt_max_ and -dP/dt_min_ were also conducted. Con, control mice without diabetes, black dots; DCM, diabetic cardiomyopathy, red squares; U0126 1mg/kg, blue triangles; Data are shown as Mean ± SEM; *p < 0.05 *vs* Con group; ^#^*p* < 0.05 *vs* DCM group. H&E, hematoxylin eosin; WGA, wheat germ agglutinin. LVEF, left ventricle ejection fraction; FS, fractional shortening; +dp/dt_max_, the maximal rate of pressure development; -dp/dt_min_, the minimal rate of pressure decay; Representative IBs of at least three independent experiments (G).

In order to confirm whether U0126 could ameliorate diabetic cardiomyopathy, we conducted animal experiments in a well-established mouse model of streptozotocin (STZ)-induced type 1 diabetes **(Figure 7A)**. 18 weeks after persistent hyperglycemia, 8 weeks U0126 (1mg/kg) or vehicle administration did not alter blood glucose in DCM mice **(Figure 7B)**. To investigate the possible protective effects of U0126 on cardiac hypertrophy, we conducted Hematoxylin and eosin (H&E) staining and WGA staining of heart tissue. We found that aberrant structural abnormalities, including disrupted cardiac fibers, deranged cellular structures, obscured intercellular border and increased cardiomyocyte transverse cross-sectional areas in diabetic model group **(Figure 7C-F)**. U0126 treatment significantly ameliorated the structural abnormalities in the hearts of diabetic mice. Consistent with the morphologic observations, the protein expression, markers of cardiac hypertrophy, were significantly increased in diabetic hearts, which were also reversed by U0126 treatment **(Figure 7G-H)**.

Before sacrifice, we evaluated the effects of U0126 on the cardiac function. Left ventricular performance was assessed by echocardiography. Compared to vehicle treated diabetic mice, mice treated with U0126 showed a significant reduction of fraction shorting and ejection fraction** (Figure 7I-K)**. We also detected the left ventricular hemodynamic alternation after administering. The result showed that diabetic mice has aggravated cardiac dysfunction, U0126 treatment decreased +dP/dt _max_ and increased -dP/dt_min_ **(Figure 7L-M)**. These data demonstrated U0126 administration exerted the beneficial effect on the left ventricle function in diabetic mice, even at a late stage.

### U0126 inhibits XBP1s' SUMOylation and increases XBP1s' nuclear translocation in diabetic mouse heart *in vivo*

**Figure 8:**
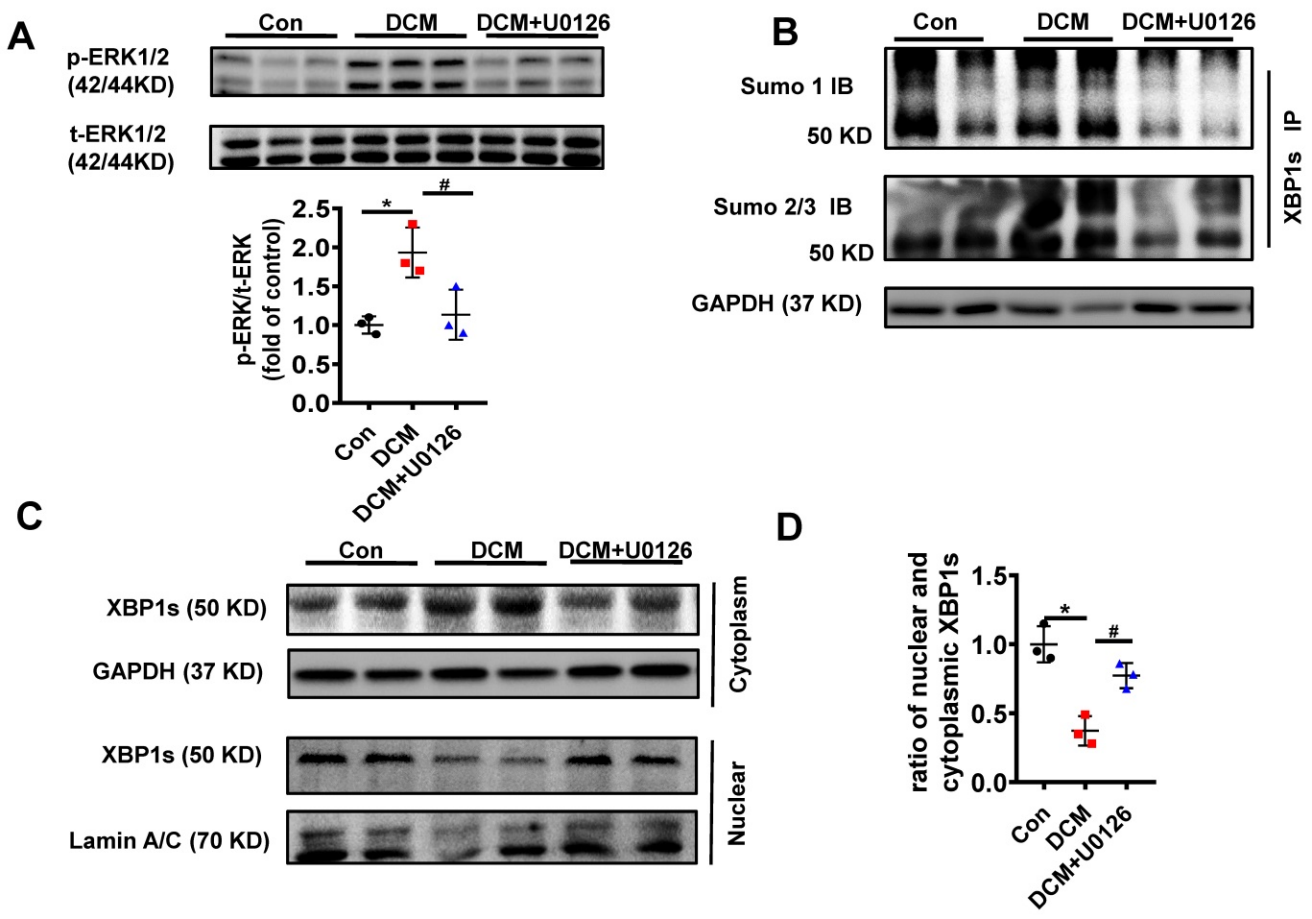
** U0126 increased XBP1s' nuclear translocation through inhibiting its SUMOylation in STZ-induced diabetic mouse heart *in vivo*.** Heart samples of each group were lysed and the extracted total proteins were processed for the detection of p-ERK1/2 and t-ERK1/2 using Western blot. **(A)** Representative immunoblots (top panel) and scatter blots (lower panel) summarizing results. **(B)** The extracted total proteins of heart samples were also immunoprecipitated with anti-XBP1s antibody, and Western blot analysis was performed with anti-SUMO1 (top panel) and anti-SUMO2/3 (lower panel) antibodies. **(C)** Representative immunoblots and **(D)** scatter blots showing nuclear and cytoplasm levels of XBP1s in the extracted proteins of each group mice. As loading controls, Lamin A/C was used for nuclear extracts and GAPDH for cytoplasm extracts. Con, control without HG, black dots; DCM: Diabetic cardiomyopathy, red squares; U0126 1mg/kg, blue triangles; Data are shown as mean ± SEM; *p < 0.05 *vs* Con group; ^#^*p* < 0.05 *vs* DCM group. IB, immunoblot; IP, immunoprecipitation. Representative IBs of at least three independent experiments (A, B and C).

To determine whether U0126 ameliorates diabetic cardiomyopathy by inhibiting XBP1s SUMOylation and increasing XBP1s nuclear translocation *in vivo*, we also determined the efficacy of U0126 on the phosphorylation of ERK1/2 in DCM mice. Consistent with the protective effects in H9C2 cells p-ERK1/2 was induced in STZ-induced in mice with DCM and U0126 markedly reversed this process **(Figure 8A)**.

Additionally, in congruent with the protective effect of U0126 in H9C2 cells *in vitro*, U0126 significantly attenuated XBP1s' SUMOylation induced by hyperglycemia in DCM mice **(Figure 8B)**. Likewise, U0126 treatment restored XBP1s' activity by promoting its nuclear translocation **(Figure 8C-D)**.

**Figure 9:**
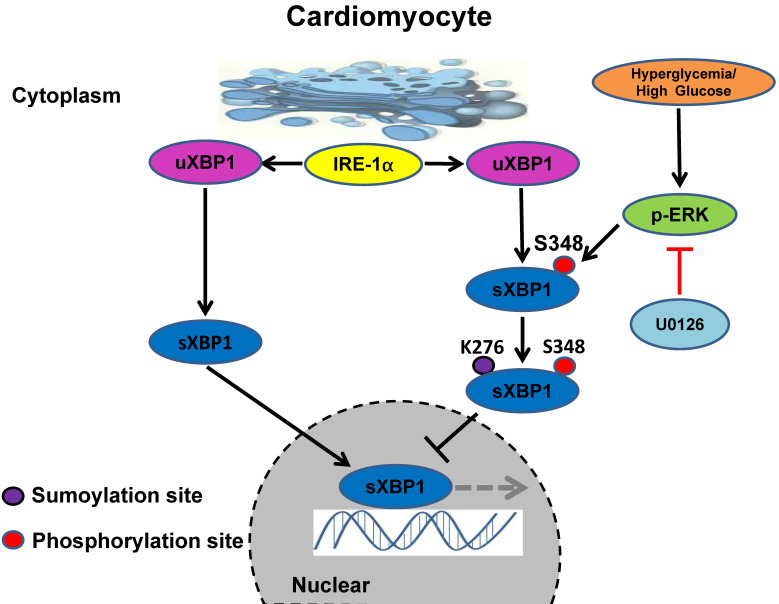
** Schematic model of the signaling pathway for XBP1s' nuclear translocation regulated by U0126 through restricting its phosphorylation and SUMOylation.** In healthy subjects the XBP1s' nuclear translocation maintains normal level. In contrast, in diabetic individuals, hyperglycemia activates Ras/MEK/ERK cascades, these activation signals lead to XBP1s Ser 348 phosphorylation, resulting in XBP1s K276 SUMOylation and subsequent impairing its nuclear translocation. U0126 could ameliorates diabetic cardiomyopathy by inhibiting Ras/MEK/ERK cascades to maintain XBP1s' nuclear translocation.

Taken together, our results identified the crucial role of ERK1/2 pathway in DCM. Chronic hyperglycemia activates Ras/MEK/ERK cascades, leading to XBP1s' Ser 348 phosphorylation, subsequently resulting in XBP1s' K276 SUMOylation and finally restricting its nuclear translocation and activity. U0126 could ameliorates DCM by inhibiting Ras/MEK/ERK cascades to maintain XBP1s' nuclear translocation **(Figure 9)**.

## Discussion

Effective therapeutic approaches are urgently needed to improve clinical outcomes of patients with DCM. However, despite intense research, the pro-pathogenic factors that can be modulated to prevent DCM remain poorly understood. In particular, the involvement of dysregulated proteostasis as result of ER stress in this process remains obscure. Our studies using a STZ induced mouse model of T1DM *in vivo* and complementary experiments in H9C2 cardiomyoblasts *in vitro* provide the following major findings: 1. XBP1s' nuclear translocation in cardiomyocyte correlates well with the DCM outcomes; 2. Chronic hyperglycemia induced activation of MAP kinase ERK1/2 can phosphorylate XBP1s on its serine residue 348, which requires for its SUMOylation on lysine residue 276 and results in its cytoplasmic retention; 3. ERK1/2 inhibitor U0126 can promote XBP1s' nuclear translocation in cardiomyocyte by restricting its phosphorylation and SUMOylation *in vitro* and *in vivo*, provides novel therapeutic avenue for DCM.

Studies showed that HF and case-fatality remains higher in T1DM than in people without diabetes mellitus, moreover T1DM patients have an even higher incidence of HF than T2DM^9^, ^10^, ^40^. Since clinical features differ between the two types of DM, underlying mechanism of DCM may be partially distinct^11^. Consistent with the hypothesis, new evidences are emerging. For instance, experimentally cardiomyocyte autophagy was enhanced in T1DM but suppressed in T2DM mouse models^41^. Clinically neither ACE-inhibitor nor statin therapy worked effectively in T1DM patients when compared to T2DM patients^42^. Therefore, DCM in T1DM is distinct from the DCM in T2DM and requires separate assessment^13^. Nevertheless, nowadays diabetes appears to be largely used as a synonym for T2DM^14^. Thus, it is prerequisite to explore the unique pathophysiological mechanisms of DCM in T1DM. Within the current study we focused on the DCM in a STZ induced T1DM mouse model and found that the key transcription factor of mammalian UPR XBP1s plays a pivotal role. In consistent with our findings XBP1s has been reported to regulate heart function in ischemia reperfusion Injury and isoproterenol-induced hypertrophy mouse models^43^, ^44^.

Diabetic cardiomyopathy is initially characterized by myocardial fibrosis, dysfunctional remodeling, and associated diastolic dysfunction, later by systolic dysfunction, and eventually by clinical heart failure^4^. In order to unveil the underlying mechanisms for this cardiac complication, many DCM animal models have been developed^45^-^47^. However, there is so far no standard protocol for establishing a T1DM DCM rodent model. Regarding STZ induced T1DM rodent model, investigators use different dosages and time points, the later range from 4 to 24 weeks^48^-^50^. Intriguingly, Gu et. al. analyzed STZ induced T1DM rat model at different time points, including 2, 12 and 24 weeks. Echocardiography analysis revealed that cardiac dysfunction is observed at 12 weeks onwards, while cardiac fibrosis and hypertrophy only became obvious at 24-week time point^50^. Albuminuria is a marker of microangiopathy and endothelial dysfunction and high level of albuminuria is associated with left ventricular dysfunction, especially in asymptomatic diabetic patients^51^, ^52^. Our previous study showed that in a T1DM diabetic nephropathy mouse model, a reduction in nuclear XBP1s associated with increased nuclear ATF6 and CHOP in kidney was apparent at 10 weeks and peaked at 26 weeks post STZ injection and coincided with the onset of albuminuria. When the hyperglycemia and albuminuria were stably induced, the mice were treated with the chemical chaperone TUDCA at 18 weeks post STZ injection to attenuate ER stress. Surprisingly, after 8 weeks treatment we observed an increase in nuclear XBP1s associated with decreased nuclear ATF6 and CHOP in kidney and alleviated albuminuria, reflecting partial disease reversal^20^. Based on these observations, we did the intervention at 18 weeks and the final analysis at 26 weeks post STZ injection in the current study. While a recent study demonstrates that STZ induced diabetic female mice exhibit a heightened susceptibility to diastolic dysfunction, despite exhibiting a lower extent of hyperglycemia than male mice^53^, we used only male mice throughout the study to avoid the gender discrepancy.

As mentioned above, we observed a positive correlation between HF indices and nuclear translocation of XBP1s in T1DM mouse hearts *in vivo* and high glucose treated H9C2 cardiomyoblasts *in vitro*. Contrarily, Wang *et al*. reported recently that in cultured H9C2 cardiomyoblasts high glucose promoted the nuclear translocation of XBP1s and CHOP expression, resulting in cardiomyocytes apoptosis *in vitro*^54^. However, we and others could show that high glucose impaired the nuclear localization of XBP1s in cultured renal epithelial cells, endothelial cells and mesangial cells *in vitro* and in kidney of diabetic animals *in vivo* as well^53^, ^55^, ^56^. Moreover, cardiomyocyte-specific overexpression of XBP1s in mice protects against ischemia-reperfusion-induced injury whereas silencing XBP1s in the heart exacerbated the injury in a hexosamine biosynthesis dependent pathway^43^. These studies indicate a protective role of nuclear XBP1s accumulation in cardiac injury and diabetic nephropathy. Besides, in mouse models of obesity nuclear translocation of XBP1s in hepatocytes could improve the glucose metabolism^57^, ^58^.

Cross-talks between various protein posttranslational modifications are critical for the regulation of coordinated networks for physiological functions^59^, ^60^. In the current study we could show that S348 phosphorylation of XBP1s is required for its K276 SUMOylation and nuclear exportation under high glucose condition in cardiomyocytes. Our results are in agreement with findings on other transcriptional factors, such as GATA-1, heat shock factors and MEF2A. Paradoxically, SUMOylation can be impaired by phosphorylation modification. For instance, MAPK can block the SUMOylation of AIB1 protein *via* its phosphorylation^59^. Moreover, there is evidence on SUMOylation dependent phosphorylation and as well^61^. As an example, the SUMOylation status of α subunit of casein kinase II can affect the phosphorylation of its substrates^59^. Interestingly, Hietakangas V *et al.* discovered a phosphorylation-dependent SUMOylation motif, which is composed of a SUMOylation consensus site and an adjacent proline-directed phosphorylation site (ΨKxExxSP)^62^. However, such motif does not exist in XBP1s, implying the existence of other possible motifs. Transcription factors SUMOylation can regulate in the intracellular localization of target proteins^63^, ^64^. Our data suggested an inhibitory effect of K276 SUMOylation on XBP1s' nuclear translocation, since a loss of function mutant K276R promoted its nuclear translocation in cardiomyocytes under high glucose condition. Future studies with cardiomyocyte specific XBP1 SUMOylation and phosphorylation mutants/knock-in mice models are required for further verification of the pathophysiological mechanisms of these post-translational modifications *in vivo*. In general, the complexity of the posttranslational modification cross-talks contributes to the fine tuning of the physiological and pathological proteomic network regulation and it necessitates further elucidation.

Numerous kinases, such as MAPKs, CKD5, AKT, IKKbeta,* etc*., have been identified to regulate the phosphorylation status of XBP1s^65^-^68^. In the current study, we found ERK1/2, a member of the MAPK family was specifically regulated in the diabetic mouse heart samples. Thus, using in-silico analysis, we obtained the potential phosphosites of ERK on XBP1s and further site directed mutagenesis study identified that S384 was crucial for its SUMOylation modification^66^. Subsequent ERK inhibitor U0126 intervention ameliorated DCM indices in mice, which implies that inhibiting phosphorylation of XBP1s could be one of the possible mechanisms. Since ERK has much diverse cellular roles and its inhibitor has potential off-target effects^69^, we could not exclude other possibilities. Hence, cardiomyocyte specific XBP1 SUMOylation and phosphorylation mutants knock in mice models would be better approaches in the future.

## Conclusion

Taken together, we have confirmed the previously unknown function of MEK/ERK pathway in regulation of ER-stress in DCM, and that was related to XBP1s' posttranslational modification cross-talks in cardiomyocyte. Targeting MEK/XBP1 pathway could be a therapeutic avenue for the treatment of DCM.

## Supplementary Material

Supplementary figures and table.

## Abbreviations

DCM: diabetic cardiomyopathy
CVD: cardiovascular disease
ER-Stress: endoplasmic reticulum stress
STZ: streptozotocin
XBP1: X-box binding protein
HF: heart failure
UPR: unfolded protein response
SENP1: Sentrin/SUMO-specific protease 1
WGA: wheat germ agglutinin
ANP: atrial natriuretic peptide
BNP: B type natriuretic peptide
β-MHC: β-myosin heavy chain
MAPKs: Mitogen-activated protein kinases
ERK1/2: extracellular signal-regulated kinase 1/2
