# Supplementary material for: The MEK inhibitor U0126 ameliorates diabetic cardiomyopathy by restricting XBP1's phosphorylation dependent SUMOylation
Source: Int J Biol Sci. 2021 Jul 13;17(12):2984–99. doi: 10.7150/ijbs.60459 (PMC8375222; doi:10.7150/ijbs.60459)
Supplement: Supplementary file 1 — Supplementary figures and table. [file ijbsv17p2984s1.pdf]

Supplementary Figure 1

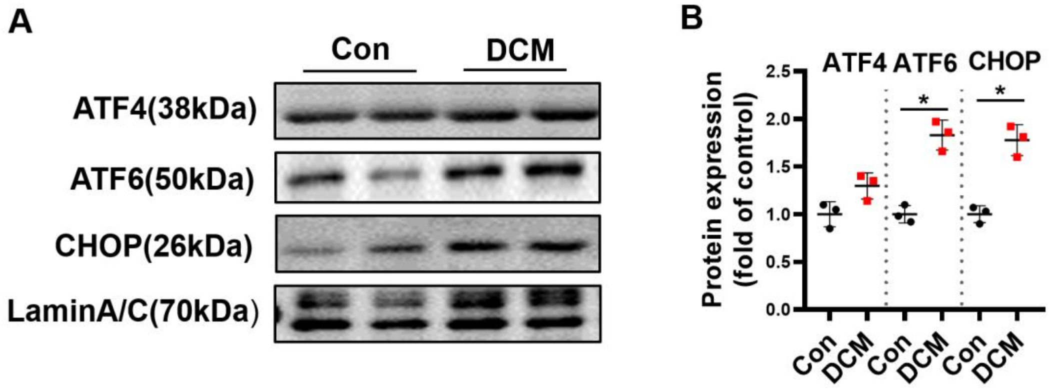

ER transcription factors were differentially regulated in diabetic mouse heart. Representative immunoblots (A) and scatter blot (B) showing nuclear levels of ER transcription factors ATF4, ATF6 and CHOP in heart samples of wild-type control (Con) and STZ-induced diabetic mice (DCM). The 50 kDa form of ATF6 is shown in (A). Lamin A/C is used as loading controls for nuclear extracts (A). (n≥6 mice per group). Con: control mice without diabetes, black dots; DCM: diabetic cardiomyopahty, red squares; Mean±SEM., **P*<0.05 (*t*-test).

Supplementary Figure 2

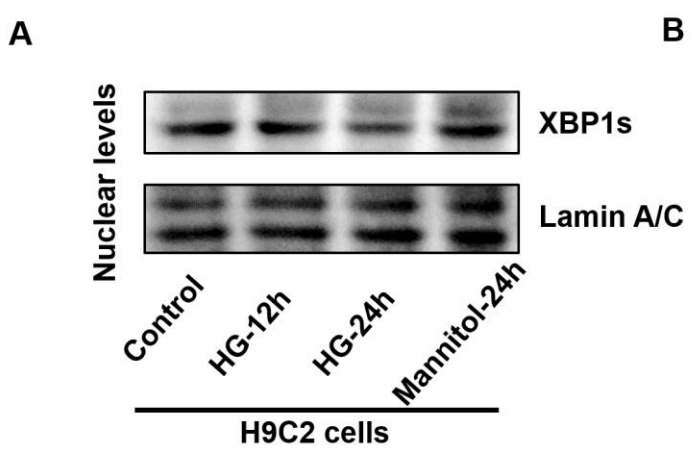

High glucose but not mannitol restrains XBP1s nuclear translocation in H9C2 cells. (A) Representative immunoblots showing nuclear levels of ER transcription factor XBP1s in H9C2 cells after treatment with HG (25mM) or mannitol (25 mM) at indicated time points. (B) scatter blot summarized the results. Con, black dots; HG-12h, red squares; HG-24h, blue triangles; Mannitol-24h, green dots. Representative immunoblots of three independent repeat experiments are shown.

Supplementary Figure 3

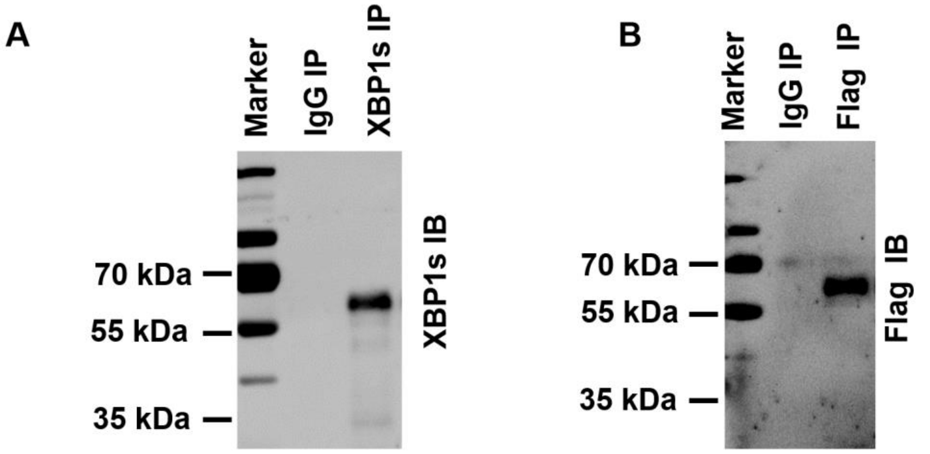

IgG controls for immunoprecipitation experiments. (A) Immunoblotting of XBP1s (XBP1s IB) following immunoprecipitation using antibodies against XBP1s or IgG control (IgG IP) from whole cell lysates of control H9C2 cells. (B) Immunoblotting of Flag (Flag IB) following immunoprecipitation using antibodies against Flag or IgG control (IgG IP) from whole cell lysates of control H9C2 cells.

Supplementary Figure 4

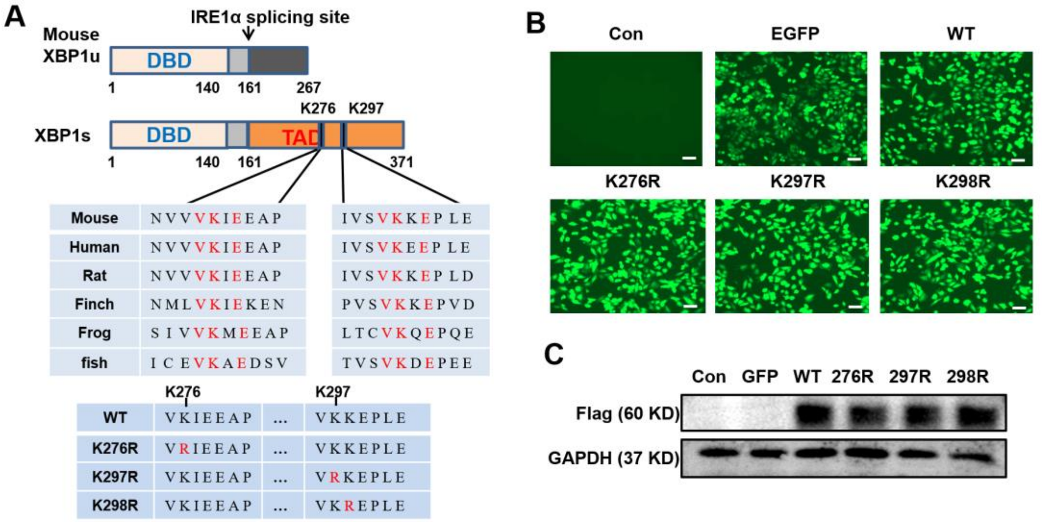

(A) Schematic diagram shown for domains in both XBP1u and XBP1s – shared regions shown with the same color. DBD, DNA binding domain. TAD, transactivation domain. Diagram of mutagenesis of lysine residues (K) to arginine (R) at the SUMO consensus motifs of mouse XBP1s protein. (B) Fluorescent microscopic images of XBP1s-EGFP fusion constructs and (C)Immunoblots of Flag-XBP1s from protein extracts in H9C2 cells upon transfection with different Flag-XBP1s adenoviruses. GAPDH served as loading controls. scale bar, 200 μm.

Supplemental Table 1: UPR gene primers for quantitative polymerase chain reaction

	Genes	Pimers	Sequences
1	Srebf2	Forward	GCCGAACTGGGCGATGGAT
		Reverse	CGTCGATGTCCCCGAGAGTC
2	Rpn1	Forward	GCCCGGTGATTGTTGCTTAC
		Reverse	AACAGGATGTAGAAGGCGGC
3	Cct7	Forward	CTTACTGCACCCTCCCAGAC
		Reverse	GGTTAGCTAAGAGGTGGCCG
4	Htra2	Forward	AGCTCCGAGAGCCAAGTTTC
		Reverse	TCCCCAATGGCCAAGATCAC
5	Creb3	Forward	AAGGCTTTTGGAGTGTGCCC
		Reverse	GGGGGCCAGGAATGTATGTT
6	Bax	Forward	AGGACGCATCCACCAAGAAG
		Reverse	CAGTTGAAGTTGCCGTCTGC
7	Nucb1	Forward	AGCAGGTGACCAGAAAGACG
		Reverse	TGAGAATCCAGCTGTGGCAG
8	Ganab	Forward	ATGCCCGTGGACTTATGGC
		Reverse	TCTGGTTGCCTTGTCACCATC
9	Canx	Forward	TCCACTTTTCAGACTGCCCC
		Reverse	ACCATTACTGGACAGAGGAAGTT
10	Erp44	Forward	ATGTGTCCCTCTTGTCCGAG
		Reverse	ACTGCCGGGCTACTTCATTC
11	Edem1	Forward	AGGCGACCAAGAATCCCTTC
		Reverse	AGAAGCTCTCCATCCGGTCT
12	Vcp	Forward	TCTCGTAGCCGTTACCCTCA
		Reverse	ACCGATTGGGACGGTTCTTC
13	Dnajb9	Forward	CGAACAGGACGAAGGTTGCT
		Reverse	ACTGACTGTGGAGTTGCCAT
14	Dnajb3	Forward	GCTGCTTCGCATTGATACCC
		Reverse	CCTATCCCTGCGATCCCAAC
15	Herpud1	Forward	GCTTCCAAAGCAGGAAAAGCG
		Reverse	TGACTAGTGTTGTCCGGCTG
16	Hspa2	Forward	TCGTCCTAACGTTGCTTTGC
		Reverse	CTGGTCGTTGGCGATGATCT
17	Rnf5	Forward	GAATGCCCGGTGTGTAAAGC
		Reverse	CGGGGTGGGGTTTTCAATCT
18	Sec62	Forward	AGCAGTGCACACTCCTTTCA
		Reverse	GCCTTTCCTCCGGACAACAT
19	Sec63	Forward	AGCAAAGACGACGAAGCAGA
		Reverse	TCAGCTCTACCTCGGGAAAGT

20	Ufd1l	Forward	ACAGGTCAGATGTGGAGAAAGG
		Reverse	ACCACAGTGTGTCATTCGGT
